# Genetic variability in drug transport, metabolism or DNA repair affecting toxicity of chemotherapy in ovarian cancer

**DOI:** 10.1186/s40360-015-0001-5

**Published:** 2015-02-27

**Authors:** Sandrina Lambrechts, Diether Lambrechts, Evelyn Despierre, Els Van Nieuwenhuysen, Dominiek Smeets, Philip R Debruyne, Vincent Renard, Philippe Vroman, Daisy Luyten, Patrick Neven, Frédéric Amant, Karin Leunen, Ignace Vergote, on behalf of the Belgian and Luxembourg Gynaecological Oncology Group (BGOG)

**Affiliations:** Division of Gynaecologic Oncology and Leuven Cancer Institute, University Hospitals Leuven, KU Leuven, Herestraat 49, 3000 Leuven, Belgium; Vesalius Research Center, VIB, Leuven, Herestraat 49, Box 912, 3000 Leuven, Belgium; Laboratory for Translational Genetics, Department of Oncology, KU Leuven, Herestraat 49, 3000 Leuven, Belgium; Oncologisch Centrum, Algemeen Ziekenhuis Groeninge, Loofstraat 43, 8500 Kortrijk, Belgium; Dienst Oncologie, Algemeen Ziekenhuis Sint Lucas, Groenebriel 1, 9000 Gent, Belgium; Dienst Medische Oncologie, Onze-Lieve-Vrouwziekenhuis, Moorselbaan 164, 9300 Aalst, Belgium; Dienst Medische Oncologie, Jessa Ziekenhuis, Stadsomvaart 11, 3500 Hasselt, Belgium

**Keywords:** Ovarian cancer, Chemotherapy, Toxicity, SNPs, Pharmacogenetics

## Abstract

**Background:**

This study aimed to determine whether single nucleotide polymorphisms (SNPs) in genes involved in DNA repair or metabolism of taxanes or platinum could predict toxicity or response to first-line chemotherapy in ovarian cancer.

**Methods:**

Twenty-six selected SNPs in 18 genes were genotyped in 322 patients treated with first-line paclitaxel-carboplatin or carboplatin mono-therapy. Genotypes were correlated with toxicity events (anemia, neutropenia, thrombocytopenia, febrile neutropenia, neurotoxicity), use of growth factors and survival.

**Results:**

The risk of anemia was increased for variant alleles of rs1128503 *(ABCB1, C > T*; p = 0.023, OR = 1.71, 95% CI = 1.07-2.71), rs363717 *(ABCA1, A > G*; p = 0.002, OR = 2.08, 95% CI = 1.32-3.27) and rs11615 (*ERCC1, T > C*; p = 0.031, OR = 1.61, 95% CI = 1.04-2.50), while it was decreased for variant alleles of rs12762549 *(ABCC2, C > G*; p = 0.004, OR = 0.51, 95% CI = 0.33-0.81). Likewise, increased risk of thrombocytopenia was associated with rs4986910 *(CYP3A4, T > C*; p = 0.025, OR = 4.99, 95% CI = 1.22-20.31). No significant correlations were found for neurotoxicity. Variant alleles of rs2073337 *(ABCC2, A > G*; p = 0.039, OR = 0.60, 95% CI = 0.37-0.98), rs1695 (ABCC1, A > G; p = 0.017, OR = 0.55, 95% CI 0.33-0.90) and rs1799793 (ERCC2, G > A; p = 0.042, OR = 0.63, 95% CI 0.41-0.98) associated with the use of colony stimulating factors (CSF), while rs2074087 *(ABCC1, G > C*; p = 0.011, OR = 2.09, 95% CI 1.18-3.68) correlated with use of erythropoiesis stimulating agents (ESAs). Homozygous carriers of the rs1799793 *(ERCC2, G > A)* G-allele had a prolonged platinum-free interval (p = 0.016).

**Conclusions:**

Our data reveal significant correlations between genetic variants of transport, hepatic metabolism, platinum related detoxification or DNA damage repair and toxicity or outcome in ovarian cancer.

**Electronic supplementary material:**

The online version of this article (doi:10.1186/s40360-015-0001-5) contains supplementary material, which is available to authorized users.

## Background

Ovarian cancer is the fifth most common cause of cancer death in women and the leading cause of gynaecological cancer-related death in the developed world [1]. Despite optimization of debulking surgery and chemotherapy regimens, the overall 5-year survival in advanced stage disease is only 29% [2]. The current standard first-line chemotherapy is a combination of paclitaxel and carboplatin. This treatment is associated with serious hematologic toxicities including grade 3–4 anemia (incidence 4.3-6.6%), grade 3–4 thrombocytopenia (4.7-12.9%), grade 3–4 neutropenia (37-89%), febrile neutropenia (2.3-8%) [3-6] and grade 2–4 peripheral neuropathies (32-36%), resulting in dose reductions, treatment delays and representing an important physical, psychological and financial burden for the patient and society. Inter-individual differences in both toxicity and outcome related to treatment with paclitaxel-carboplatin are reported. A few patient-related risk factors for toxicity have been identified, such as elderly age (≥65 years), poor performance status and poor nutritional status [7]. Furthermore, tumor-related factors including advanced stage at diagnosis, high-grade serous disease and residual tumor after debulking surgery are associated with poor survival. Genetic variability represents another potential factor explaining this inter-individual variability.

Genes related to drug transport, metabolism, detoxification and DNA repair could influence the cytotoxic effects associated with chemotherapy, including those involved in the transport (e.g., *ABCB1*, *ABCC1*, *ABCC2*, *ABCG2*, and *SLCO1B3*) [8-24], hepatic metabolism (*CYP3A4*, *CYP3A5*, *CYP2C8*, *CYP1B1*) [8-12,14,18,25-27] and pharmacodynamics (e.g., *MAPT*, *TUBB*, *TP53*) [28,29] of paclitaxel. Likewise, genes involved in detoxification (e.g., *GSTP1*, *GSTT1*, *GSTM1*) [30-33] and base-excision DNA repair (e.g., *ERCC1*, *ERCC2*, *XRCC1*) [34,35] have previously been linked with cytotoxicity of platinum agents. In particular, genetic variants in these genes, which generally are supposed to reduce the function of the affected gene, have been proposed to underlie the inter-individual differences in chemotherapy related hematologic and neurotoxicity. Likewise, variants in other genes, including *SLC12A6*, *SERPINB2*, *PPARD* and *ICAM* have been proposed to contribute to chemotherapy-induced peripheral neurotoxicity [36]. Most studies identifying these candidate genes, however, have been performed in small study populations and were limited to testing only a few variants. Consequently, most of the reported associations have failed to be replicated in subsequent large-scale validation studies. Furthermore, most studies did not correlate genotypes with detailed clinical toxicity data.

In the current study, we therefore aimed to assess prior associations for 26 selected genetic variants in 18 genes, in a large cohort of 322 ovarian cancer patients treated with paclitaxel-carboplatin combination therapy or carboplatin mono-therapy of whom detailed clinical toxicity data were available.

## Methods

### Study population

All ovarian cancer patients presenting in participating hospitals of the Belgian and Luxembourg Gynaecological Oncology Group (BGOG) were recruited for this study. Collection of germ-line DNA and baseline patient characteristics were collected for each patient. Disease characteristics were recorded after histologic examination with registration of tumor stage according to the International Federation of Gynecology and Obstetrics (FIGO) classification, residual disease after debulking surgery, measurement of tumor size on computed tomography (CT) scans and determination of cancer antigen 125 (CA125) before, during and after chemotherapy. Response to treatment and disease progression were evaluated based on radiologic examination according to the Response Evaluation Criteria in Solid Tumors Group (RECIST) criteria [37]. Paclitaxel was administered at a starting dose of 175 mg/m^2^ and carboplatin at a starting area under the plasma concentration-versus time curve (AUC) of 5–7 mg/ml/min, with possible dose reductions after the occurrence of severe toxicity. During treatment, the use of erythropoiesis stimulating agents (ESAs) and colony stimulating factors (CSFs) was conform to uniform institutional standards; ESAs are given during treatment with chemotherapy in symptomatic patients with a hemoglobin level below 11 g/dl while CSFs are given if (1) neutropenia grade 4 (ANC <500/mm^3^) together with fever > 38°C *or* (2) neutropenia grade 4 (ANC < 500/mm^3^) during minimum 5 consecutive days. Toxicity during chemotherapy was systematically and routinely scored according to the Common Terminology for Adverse Events (CTCAE) version 4.0. Hematological toxicity was scored based on routinely performed weekly complete blood counts during treatment and before each cycle to determine the nadir of anemia, neutropenia and thrombocytopenia of each administered cycle, neurotoxicity was scored at each clinical-physical examination before each cycle. The scored toxicities for each patient together with all events of neutropenic fever and use of growth factors were systematically recorded in medical electronic records and for the purpose of the present study retrospectively collected by two independent investigators. The highest grade of toxicity over all courses within a patient was reported, if weekly performed blood counts were not available for each administered cycle or if neurotoxicity was not scored for every cycle, the patient was excluded from the analysis. The primary objective of this study was the correlation of genetic variation with the occurrence of hematologic toxicity or neurotoxicity in patients treated with first-line carboplatin with or without paclitaxel. Secondary objectives included the relation between genetic variation and the need for growth factors during treatment with chemotherapy, platinum-free interval (PFI) defined as the time between the last first-line platinum dose and progression, and overall survival (OS). Analyses for PFI and OS were performed in the population receiving a combination of carboplatin and paclitaxel (n = 266) with exclusion of the more favorable prognostic population receiving carboplatin alone, based on clinical prognostic parameters such as FIGO stage, tumor grade and histological subtype. All included patients provided written informed consent before enrollment. The Medical Ethics Committee of the Leuven University Hospitals approved the study (ML6541), serving as central site with the authority to approve the study for all participating sites.

### Genotyping

We performed an extensive literature search before start of enrolment to identify genes associated with treatment outcome or toxicity after platinum and/or paclitaxel administration [8-34,36]. We then selected common missense or synonymous mutations in these genes, as well as a number of SNPs that were located in the promoter region of these genes, but have previously been correlated with toxicity after platinum. In addition, we selected 5 additional SNPs previously associated with thalidomide-related neuropathy to investigate their role in repair mechanisms and inflammation in the peripheral nervous system leading to altered neurotoxicity, rather than having a thalidomide-specific contribution to correlated neurotoxicity [36]. Genomic DNA was extracted from the leucocyte fraction of whole blood samples (Qiagen DNeasy blood and tissue kit). All selected SNPs were genotyped using Sequenom MassARRAY technology (Sequenom Inc., CA, USA), as reported previously [38]. Overall 26 SNPs in 18 genes (Table 1) were genotyped with an individual call rate >95% and an overall success rate >98.5%. We genotyped 15 duplicate samples revealing a genotype accuracy exceeding 99%.

**Table 1:** **Overview of the 26 genotyped single nucleotide polymorphisms (SNPs)**

**Gene**	**Name**	**Function of the gene product**	**Variant allele (rs number, nucleotide, amino acid change)**	**Effect of the polymorphism on the toxicity or clinical outcome**
ABCB1	Multidrug resistance 1, P-glycoprotein	ATP binding membrane transporter implicated in efflux of cytotoxic agents	rs1128503, c.1236C>T, Gly412Gly	Homozygous carriers of the variant allele: docetaxel clearance decreased [9].
			rs1045642, c.3435C>T, Ile1145Ile	Variant allele carriers: more pronounced neutrophil depression following treatment with paclitaxel ± carboplatin [18] and increased AUC of the paclitaxel metabolite 3′-p-hydroxypaclitaxel [8].
				Homozygous carriers of the variant allele: decreased risk of neutropenia and neurotoxicity [11]
				No correlation was found with pharmacokinetics, toxicity or outcome in OC patients in different other studies [9,10,12,13,15,17].
			rs2229109,c.1199G>A, Ser400Asn	Variant allele carriers: correlation with *in vitro* resistance to paclitaxel [22].
ABCC1	Multidrug resistance-associated protein 1	ATP binding membrane transporter implicated in efflux of cytotoxic drugs	rs2230671, c.4002G>A, Ser1334Ser	In vitro evidence: over-expression of ABCC1 protein has been associated with a low degree of resistance to paclitaxel [23].
			rs2074087, c.2284-30G>C	No correlation of variants in rs2230671 and rs2074087 with toxicity and outcome after platinum/taxane treatment in OC patients [12].
ABCC2	Multidrug resistance-associated protein 2	ATP binding membrane transporter implicated in efflux of cytotoxic drugs	rs2073337, c.1668+148A>G	In vitro evidence: paclitaxel and docetaxel are ABCC2 substrates in cell lines [24]. No correlation was found with toxicity or treatment outcome with platinum-taxane treatment in OC patients [12,17].
			rs12762549, g.101620771C>G	Variant allele carriers from Japan: increased risk for severe neutropenia following treatment with docetaxel [19].
ABCG2	ATP-binding cassette sub-family G member 2	ATP binding membrane transporter implicated in efflux of cytotoxic drugs	rs2231142, c.421C>A, Gln141Lys	Variant allele carriers in OC: 6-month longer median PFS following platinum/taxane-based chemotherapy [17].
ABCA1	ATP-binding cassette sub-family A member 1	ATP binding membrane transporter, efflux pump for S1P and cholesterol	rs363717, c.*1896 A>G	Variant allele carriers: decreased risk on thalidomide related neuropathy grade ≥2 [36].
SCLO1B3	Solute carrier organic anion transporter family member 1B3	Hepatocyte membrane transporter involved in the transport of cytotoxic drugs	rs4149117, 334T>G, Ser112Ala	Docetaxel and paclitaxel transport by SCLO1B3-expressing oocytes was higher compared to controls *in vitro* [20].
			rs11045585, c.1683-5676A>G	Variant allele carriers from Japan: increased docetaxel induced leukopenia/neutropenia [19], higher docetaxel clearance and lower AUC in nasopharyngeal carcinoma patients [21].
CYP1B1	Cytochrome P450 family 1, subfamily B, polypeptide 1	Enzyme in the oxidative metabolic pathway of exogenous chemicals including taxanes and estrogens	rs1056836, 4326C>G, Val432Leu (CYP1B1*3)	Homozygous carriers of the wild-type allele: decreased risk of grade 3/4 gastro-intestinal toxicity in docetaxel treated OC patients in the development but not in the validation set [12].
CYP3A4	Cytochrome P450, family 3, subfamily A, polypeptide 4	Enzyme in the oxidative metabolic pathway of exogenous chemicals including taxanes and estrogens	rs2740574, g.135607G>A (CYP3A4*1B)	CYP3A4 activity determined the dominant metabolic pathway for paclitaxel [14].
				Homozygous carriers of the variant allele: decreased clearance of docetaxel [26].
				Homozygous carriers of the variant allele: increased risk of invasive OC [27].
			rs4986910, c.1331T>C, Met444Thr (CYP3A4*3)	No correlation with pharmacokinetics, toxicity or outcome in OC patients treated with carboplatin + paclitaxel or docetaxel [9,10,12].
CYP3A5	Cytochrome P450, family 3, subfamily A, polypeptide 5	Enzyme in the oxidative metabolic pathway of exogenous chemicals including taxanes and estrogens	rs776746, c.219-237G>A	Homozygous carriers of the variant allele: increased neurotoxicity following paclitaxel treatment ^25^. No correlation with pharmacokinetics, toxicity or outcome in OC patients treated with carboplatin + paclitaxel or docetaxel [9,10,12].
TP53	Tumor protein 53	Transcription factor regulating multiple cellular functions, critical for maintenance of genomic stability	rs1042522, c.215C>G, Pro72Arg	Associated with a small increase in risk of OC [29], twofold increased risk of OC in proline carriers and a longer progression-free survival in homozygous arginine allele carriers [28]. Homozygous carriers of the variant allele: increased severity of neutropenia [32].
MAPT	Microtubule-associated protein tau	Protein stimulating tubulin polymerization, stabilizing microtubules	rs11568305, c.215C>G, Pro587=	No correlation with toxicity or outcome in OC patients treated with carboplatin + paclitaxel or docetaxel [12].
GSTP1	Gluthathione S-transferase pi	Xenobiotic enzyme involved in the prevention of platinum-based DNA damage	rs1695, c.313A>G, Ile105Val	Variant allele carriers: decreased oxaliplatin-related neuropathy [30], decreased docetaxel-induced grade 2 neuropathy [31], decreased risk of hematologic toxicity [15].
			rs1138272, c.341 C>T, Ala114Val	Variant allele carriers compared to homozygous carriers of the wild-type allele: decreased PFS following cisplatin-gemcitabine [32].
				In other studies, no association with toxicity in OC patients [12,32].
ERCC1	Excision repair cross complementation group1	Enzyme involved in nucleotide excision repair of DNA	rs11615, c.354T>C, Asn118Asn	Variant allele carriers: decreased platinum resistance [34].
			rs3212961, 17677G>T	Variant allele carriers compared to homozygous carriers of the wild-type allele: increased risk on severe neutropenia and increased likelihood of overall survival following cisplatin-gemcitabine [32].
				No correlation for both genetic variants with toxicity/outcome for OC patients [12].
ERCC2	Excision repair cross complementation group2	Enzyme involved in nucleotide excision repair of DNA	rs1799793, c.934G>A, Asp312Asn	Variant allele carriers: increased severity of neutropenia in OC patients receiving cisplatin-cyclophosphamide [33].
SLC12A6	Solute carrier family 12 member 6	Integral membrane protein that lowers intracellular chloride concentrations	rs7164902,g.34551082G>A, Leu144Leu	Variant allele carriers: decreased risk on thalidomide related neuropathy grade ≥2 [36].
SERPINB2	Serpin peptidase inhibitor B member 2	Inhibitor of urokinase plasminogen activator, mediating neuro-inflammation	rs6104, 1238C>G, Ser413Cys	Variant allele carriers: decreased risk on thalidomide related neuropathy grade ≥2 [36].
PPARD	Peroxisome proliferator-activated receptor delta	Nuclear receptor protein playing a role in neuro-inflammation	rs2076169, T>C	Variant allele carriers: decreased risk on thalidomide related neuropathy grade ≥2 [36].
ICAM1	Intercellular Adhesion Molecule 1	Cell surface glycoprotein in endothelial and immune system cells	rs1799969, 241G>A	Variant allele carriers: decreased risk on thalidomide related neuropathy grade ≥2 [36].

The following 7 genetic variants failed genotyping: rs2032582 (Ser893Ala in ABCB1), rs2273697 (Val417Ile in ABCC2), rs1058930 (Ile194Met in CYP2C8), rs11572080 (Arg69Lyes in CYP2C8), rs10509681 (Lys329Arg in CYP2C8), rs12721627 (Thr185Ser in CYP3A4), rs25487 (Gln398Arg in XRCC1). Rs6103 was replaced by rs6104 because these were in full linkage disequilibrium (r^2^ = 1.0). OC: ovarian cancer, NSCLS: non-small-cell lung carcinoma.

### Statistical analysis

We calculated median values and inter-quartile ranges for all continuous variables, while frequencies and percentages were calculated for categorical variables. Genotype frequencies were tested for Hardy-Weinberg equilibrium using a 1°-of-freedom *χ*2-test and considered significant at P < 0.05. Each of the variants were correlated with toxicity events (i.e., the primary objective) using binary logistic regression, while assuming an additive genotypic model. Per-allele odds ratios (OR) and their respective 95% confidence intervals (CI) are reported. Regression analyses were performed without correction for covariates and after correction for relevant covariates, including age and BMI at the time of treatment, dose of carboplatin per cycle (AUC), number of administered cycles and treatment regimen (paclitaxel/carboplatin versus carboplatin alone). For anemia, an additional covariate was included, i.e., use of ESAs, whereas for neutropenia and febrile neutropenia, use of CSFs was included as an additional covariate. Secondary objectives, PFI and OS, were analyzed for 26 variants using Cox-regression analysis, adjusted for age at diagnosis only or fully adjusted for age at diagnosis, FIGO stage, tumor grade, tumor histology and residual disease after debulking surgery and PFS and OS estimates were calculated using Kaplan-Meier method. Additionally, we investigated which of the variants could predict the need for ESAs or CSFs during treatment. All tests were two-sided and statistical significance was set at p = 0.05. The Bonferroni p-value threshold correcting for the multiple testing of 26 SNPs was p < 0.0019. Statistical analyses were performed using SPSS version 19 (SPSS for Windows, Rel. 19.0.0. 2010. Chicago, Illinois, USA: SPSS Inc.)

## Results

### Study population

Between January 2009 and December 2011 (pre-specified period of 2 years), we recruited 322 ovarian cancer patients treated with 3–6 cycles paclitaxel-carboplatin combination therapy (n = 266) or 3–6 cycles carboplatin mono-therapy (n = 56) (Additional file 1: Figure S1). Of all recruited patients, 99% was Caucasian (Table 2). Hematological toxicity was analyzed in 290 patients, after exclusion of patients for which weekly blood examinations were not available (n = 32). For neurotoxicity, 56 patients treated with carboplatin monotherapy were excluded since the incidence of sensory neuropathy was significantly lower in this population (p < 0.001). One patient with pre-existing sensory neuropathy before start of chemotherapy was additionally excluded, bringing the total number of patients eligible up to 265. For the secondary objectives, PFI and OS, all patients treated with paclitaxel-carboplatin (n = 266) were included. Patient, disease and toxicity characteristics are summarized in Table 2. Briefly, grade 3–4 anemia was present in 57 patients (19.7%), grade 3–4 thrombocytopenia in 57 patients (19.7%), grade 4 neutropenia in 202 patients (69.7%), whereas only 23 patients (7.9%) presented with grade 3–4 febrile neutropenia. In the group of patients selected for neurotoxicity analysis, 48 patients (18.1%) developed grade 2–3 sensory and none motor neuropathy following combination treatment with paclitaxel-carboplatin. Minor allele frequencies (MAF) were similar to those reported previously in Caucasians and adhered to Hardy-Weinberg equilibrium. Allele frequencies of all genotyped SNPs are shown in Additional file 2: Table S1.

**Table 2:** **Patient and disease characteristics, hematologic and neuro-toxicity characteristics**

**Patient and disease characteristics**					
	**Total Number of patients recruited**	**Population for hematologic analysis**			**Population for outcome**
	**N=322**	**All patients**	**Paclitaxel-Carboplatin**	**Carboplatin**	**N=266**
		**N=290**	**N=240**	**N=50**	
**Age at diagnosis (years)**		p=0.188*		*p=0.218* ^*§*^	p=0.128*
Median	60	59	59	56	59
Range	(20-85)	(20-85)	(21-82)	(20-85)	(21-84)
**Body mass index (BMI)**		p=0.863*		*p=0.063* ^*§*^	p=0.055*
Median	25	25	24	26	24
Range	(16-39)	(16-39)	(16-39)	(18-37)	(16-39)
**Race**		p=0.951*		*p=0.517* ^*§*^	p=0.520*
Caucasian	319 (99%)	287 (99%)	238 (99%)	49 (98%)	264 (99%)
African	1 (<1%)	1 (<1%)	0 (0%)	1 (<1%)	0 (0%)
Asian	1 (<1%)	1 (<1%)	1 (<1%)	0 (0%)	1 (<1%)
Mixed: Asian-Indo-European	1 (<1%)	1 (<1%)	1 (<1%)	0 (0%)	1 (<1%)
**Histologic subtype**		p=0.532*		*p<0.001* ^*§*^	p<0.001*
Serous	258 (80%)	230 (79%)	209 (87%)	21 (42%)	231 (87%)
Mucinous	20 (6%)	19 (7%)	5 (2%)	14 (28%)	6 (2%)
Endometrioid	13 (4%)	13 (4%)	5 (2%)	8 (16%)	5 (2%)
Clear cell	17 (5%)	14 (5%)	8 (3%)	6 (12%)	11 (4%)
Mixed cell	8 (3%)	8 (3%)	7 (3%)	1 (2%)	7 (3%)
Other epithelial ovarian cancer	3 (1%)	3 (1%)	3 (1%)	0 (0%)	3 (1%)
Non-epithelial	3 (1%)	3 (1%)	3 (1%)	0 (0%)	3 (1%)
**FIGO stage**		p=0.645*		*p<0.001* ^*§*^	p<0.001*
I	55 (15%)	52 (18%)	11 (5%)	41 (82%)	14 (5%)
II	17 (5%)	15 (5%)	13 (5%)	2 (4%)	15 (6%)
III	196 (61%)	175 (60%)	169 (70%)	6 (12%)	184 (69%)
IV	54 (17%)	48 (17%)	47 (20%)	1 (2%)	53 (20%)
**Tumor grade**		p=0.235*		*p<0.001* ^*§*^	p<0.001*
1	23 (7%)	23 (8%)	13(5%)	10 (20%)	13 (5%)
2	50 (16%)	45 (16%)	30 (12%)	15 (30%)	35 (13%)
3	249 (77%)	222 (77%)	197 (82%)	25 (50%)	218 (82%)
**Residual disease**		p=0.120*		*p<0.001* ^*§*^	p=0.424*
No macroscopic disease	267 (83%)	246 (85%)	200 (83%)	46 (92%)	218 (82%)
Macroscopic disease < 1cm	7 (2%)	6 (2%)	6 (3%)	0 (0%)	7 (3%)
Macroscopic disease > 1 cm	8 (3%)	6 (2%)	6 (3%)	0 (0%)	8 (3%)
Macroscopic disease, size unknown	5 (2%)	4 (1%)	3 (1%)	1 (2%)	4 (2%)
Macroscopic disease, inoperable	35 (11%)	28 (10%)	25 (10%)	3 (6%)	29 (11%)
**Hematologic toxicity characteristics**					
		**All patients**	**Paclitaxel-Carboplatin**		**Carboplatin**
		**(N= 290)**	**(N=240)**		**(N=50)**
**Number of cycles administered**					*p=0.266* ^*§*^
**<6**		15 (5%)	14 (6%)		1 (2%)
**6**		275 (95%)	226 (94%)		49 (98%)
**Grade anemia**					*p=0.118* ^*§*^
**0/1**		62 (21%)	51 (21%)		11 (22%)
**2**		171 (59%)	136 (57%)		35 (70%)
**3**		51 (18%)	48 (20%)		3 (6%)
**4**		6 (2%)	5 (2%)		1 (2%)
**Use of Erythropoiesis stimulating Agent (ESA)**					*p=0.073* ^*§*^
**No**		220 (76%)	187 (78%)		33 (66%)
**Yes**		70 (24%)	53 (22%)		17 (34%)
**Grade neutropenia**					*p<0.001* ^*§*^
**0/1**		19 (7%)	9 (4%)		10 (20%)
**2**		14 (5%)	3 (1%)		11 (22%)
**3**		55 (19%)	32 (13%)		23 (46%)
**4**		202 (70%)	196 (82%)		6 (12%)
**Febrile neutropenia**					*p=0.740* ^*§*^
**0**		267 (92%)	217 (90%)		50 (100%)
**3**		22 (8%)	22 (9%)		0 (0%)
**4**		1 (<1%)	1 (<1%)		0 (0%)
**Use of colony stimulating factor (CSF)**					*p<0.001* ^*§*^
**No**		228 (79%)	178 (74%)		50 (100%)
**Yes**		62 (21%)	62 (26%)		0 (0%)
**Grade Trombocytopenia**					*p=0.089* ^*§*^
**0/1**		180 (62%)	156 (65%)		24 (48%)
**2**		53 (18%)	41 (17%)		12 (24%)
**3**		43 (15%)	31 (13%)		12 (24%)
**4**		14 (5%)	12 (5%)		2 (4%)
**Neurotoxicity characteristics**					
		**Population for neurotoxicity analysis (Paclitaxel-Carboplatin) (N=265)**		**Population excluded for neurotoxicity (Carboplatin) (N=56)**	
**Number of cycles administered**				*p=0.596* ^*#*^	
**<6**		18 (7%)		2 (4%)	
**6**		247 (93%)		54 (96%)	
**Grade peripheral sensory neuropathy**				*p<0.001* ^*#*^	
**0**		109 (41%)		48 (86%)	
**1**		108 (41%)		6 (11%)	
**2**		39 (15%)		1 (2%)	
**3**		9 (3%)		0 (0%)	
**Grade motor neuropathy**				*p=0.461* ^*#*^	
**0**		56 (100%)		254 (96%)	
**1**		0 (0%)		10 (4%)	
**2**		0 (0%)		1 (<1%)	
**3**		0 (0%)		0 (0%)	
**4**		0 (0%)		0 (0%)	

*: p-value calculated against the total population (n = 322), ^**§**^: p-value calculated against the population for hematologic analysis treated with taxol-carboplatin (n = 240), ^**#**^: p-value calculated against the population for neurotoxicity treated with taxol-carboplatin (n = 265).

### Association with anemia, thrombocytopenia, neutropenia and sensory neuropathy

Among the 290 patients eligible for the hematological toxicity analysis, we observed significant associations for 5 variants (Table 3). In particular, rs1128503 (*ABCB1, C > T),* rs12762549 (*ABCC2, C > G),* rs363717 (*ABCA1, A > G)* and rs11615 (*ERCC1, T > C*) were significantly associated with grade 3–4 anemia (p = 0.035, OR 1.58; p = 0.005, OR 0.55; p = 0.001, OR 1.31 and p = 0.024, OR = 1.58). After correction for relevant covariates (as explained in the statistical methods), these variants were still significantly associated with toxicity (p = 0.023, OR 1.71; p = 0.004, OR 0.51; p = 0.002, OR 2.08; and p = 0.031, OR 1.61 respectively). Another variant rs4986910 (*CYP3A4, T > C)* correlated with thrombocytopenia grade 3–4, before and after correction for relevant covariates (p = 0.012, OR 5.61 and p = 0.025, OR 4.99 respectively; Table 3). When correlating each of the variants with grade 4 neutropenia and febrile neutropenia, we did not observe a significant association. Finally, we also correlated each of the variants to sensory neuropathy in the population that was eligible for neurotoxicity analyses, but failed to identify significant associations. None of the observed associations with hematologic toxicity survived correction for multiple testing.

**Table 3:** **Association between genetic variants and hematologic toxicity**

**3A: Significant correlations with anemia**							
	**All patients N = 290 (%)**	**Patients with anemia gr 3–4 N = 57 (19.6%)**	**Patients without anemia gr 3–4 N = 233 (80.3%)**	**Unadjusted OR (95%CI)**	*p-value	**Adjusted OR (95% CI)**	**Corrected p value
**ABCB1 rs1128503**	CC 94 (32.4)	13 (22.8)	81 (34.8)	1.58 (1.03; 2.42)	0.035	1.71 (1.07; 2.71)	0.023
	CT 147 (50.7)	30 (52.6)	117 (50.2)				
	TT 49 (16.9)	14 (24.6)	35 (15.0)				
**ABCC2 rs12762549**	CC 80 (27.6)	25 (43.8)	55 (23.6)	0.55 (0.36; 0.83)	0.005	0.51 (0.33; 0.81)	0.004
	CG132 (45.5)	22 (38.6)	110 (47.2)				
	GG 76 (26.2)	10 (17.5)	66 (28.3)				
**ABCA1 rs363717**	AA 86 (29.6)	10 (17.5)	76 (32.6)	1.31 (1.98; 2.99)	0.001	2.08 (1.32; 3.27)	0.002
	GA 131 (45.2)	23 (40.3)	108 (33.5)				
	GG 73 (25.2)	24 (42.1)	49 (15.2)				
**ERCC1 rs11615**	TT 133 (45.9)	18 (31.6)	115 (49.3)	1.58 (1.06-2.35)	0.024	1.61 (1.04-2.50)	0.031
	TC 114 (39.3)	28 (49.1)	86 (36.9)				
	CC 42 (14.5)	11 (19.3)	31 (13.3)				
**3B: Significant correlations with thrombocytopenia (TCP)**							
	**All patients**	**Patients with TCP gr 3 – 4**	**Patients without TCP gr 3 – 4**	**Unadjusted OR**	*p-value	**Adjusted OR**	**Corrected p value
	**N = 290 (%)**	**N = 57 (19.6%)**	**N = 233 (80.3%)**	**(95%CI)**		**(95% CI)**	
**CYP3A4 rs4986910**	TT 280 (96.5)	51(89.5)	229(98.3)	5.61 (1.46; 21.64)	0.012	4.99 (1.22; 20.31)	0.025
	CT 9 (3.1)	5(8.8)	4(1.7)				
	CC 0 (0)	0	0				

OR: Odds Ratio using wild type as reference category. *Uncorrected p values were calculated using binary logistic regression without correction for covariates. Per-allele ORs and 95% CIs are shown. There were missing genotypes for rs12762549 (n = 2), rs11615 (n = 1) and rs4986910 (n = 1). **Corrected p values were obtained using a logistic regression for the presence or absence of anemia/thrombocytopenia/febrile neutropenia while including the following covariates: genetic variant, age, BMI, AUC of carboplatin, number of administered cycles, and use of ESA for anemia or use of CSF for febrile neutropenia. In the regression for anemia, age, BMI, administered AUC of carboplatin or number of administered cycles were not identified as significant covariates (p = 0.576, p = 0.614 and p = 0.317, p = 0.481), whereas use of ESA was significant (p = 0.034). In the regression for grade 3–4 thrombocytopenia, age and AUC of administered carboplatin were a significant covariate (p = 0.023 and p = 0.014), but BMI or number of administered cycles were not (p = 0.571 and p = 0.243). In the regression for grade 4 neutropenia, BMI and age were significant covariates (p = 0.043 and p = 0.041), while administered AUC and number of administered cycles were not (p = 0.607 and p = 0.321).

### Association between genetic variants and use of growth factors

The use of ESAs or CSFs was also correlated with each of the 26 variants to examine whether they could predict the need for an ESA or CSF during treatment with chemotherapy in ovarian cancer. After correction for relevant covariates, a significant correlation for rs2074087 (*ABCC1, G > C)* and the use of ESA was noticed (p = 0.011, OR 2.09, Table 4). After correction for covariates, CSF use was significantly correlated with rs2073337 (*ABCC2, A > G)*, rs1695 (*GSTP1, A > G)* and rs1799793 (*ERCC2, G > A)* (p = 0.039, OR 0.60; p = 0.017, OR 0.55; and p = 0.042, OR 0.63 respectively). None of the observed associations with use of growth factors survived correction for multiple testing.

**Table 4:** **Association between genetic variants and erythropoiesis stimulating agents (ESA) or colony stimulating factor (CSF) use**

**4A: Significant correlations with ESA use**							
	**All patients**	**Patients with ESA use**	**Patients without ESA use**	**Unadjusted OR (95%CI)**	*p-value	**Adjusted OR (95% CI)**	**Corrected p value
	**N = 290 (%)**	**N = 70 (24.2%)**	**N = 219 (75.8%)**				
**ABCC1 rs2074087**	GG 215 (74.4)	46 (65.7)	169 (77.2)	1.78 (1.03- 3.08)	0.054	2.09 (1.18 - 3.68)	0.011
	GC 69 (23.9)	22 (31.4)	47 (21.5)				
	CC 5 (1.7)	2 (2.8)	3 (1.4)				
**4B: Significant correlations with CSF use**							
	**All patients**	**Patients with CSF use**	**Patients without CSF use**	**Unadjusted OR**	*p-value	**Adjusted OR**	**Corrected p value
	**N = 290 (%)**	**N = 62 (21.4%)**	**N = 228 (78.6%)**	**(95%CI)**		**(95% CI)**	
**ABCC2 rs2073337**	AA 101 (34.8)	27 (43.5)	74 (32.5)	0.61 (0.39- 0.96)	0.031	0.60 (0.37-0.99)	0.039
	AG 148 (51.0)	31 (50.0)	117 (51.3)				
	GG 41 (14.1)	4 (6.5)	37 (16.2)				
**GSTP1 rs1695**	AA 121 (41.7)	33 (53.2)	88 (38.6)	0.54 (0.34; 0.86)	0.010	0.55 (0.33-0.90)	0.017
	AG 137 (47.2)	27 (43.5)	110 (48.2)				
	GG 32 (11.0)	2 (3.2)	30 (13.2)				
**ERCC2 Rs1799793**	GG 136 (48.2)	21 (33.9)	115 (50.4)	0.67 (0.45- 1.00)	0.048	0.63 (0.41-0.98)	0.042
	GA 111 (39.4)	30 (48.4)	81 (35.5)				
	AA 35 (12.4)	9 (14.5)	26 (11.4)				

OR: Odds Ratio using wild type as reference category. *Uncorrected p values were calculated using binary logistic regression for the need for ESA/CSF use without correction for covariates. Per-allele ORs and 95% CIs are shown. There were missing genotypes for rs2074087 (n = 1), rs1799793 (n = 8). **Corrected p values were obtained using a logistic regression for the need for ESA/CSF use while including the following covariates: genetic variants, age, BMI, dosage of carboplatin (AUC) and number of administered cycles.

### Effects of variants on PFI and OS

**Figure 1:**
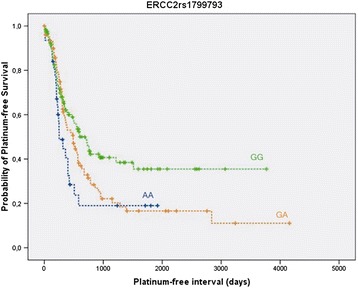
**Kaplan-Meier curve for platinum-free interval correlated with polymorphisms of rs1799793 in ERCC2.** Kaplan-Meier survival analysis reveales a significant advantage in PFI for GG cariers of rs1799793 compared to AA or GA carriers (p = 0.016).

The median follow-up of all patients participating to the study was 2.5 years (95% CI = 2.2-2.8 years) with 157 events for progression (59%) and 84 events for OS (31.6%). Uncorrected P-values were calculated using Cox regression analysis either adjusted for age at diagnosis only or fully adjusted for age at diagnosis, FIGO stage, tumor grade, tumor histology and residual disease after debulking surgery. Only one variant, rs1799793 (*ERCC2 G > A)*, was significantly correlated with PFI in both cases (p = 0.003, HR = 0.71, 95% CI = 0.57-0.89, p = 0.016, HR = 0.75, 95% CI = 0.60-0.95). In particular, Kaplan-Meier survival analysis revealed a significant advantage in PFI for GG carriers of rs1799793 compared to AA or GA carriers (p = 0.016; Figure 1). Variants rs12762549 (*ABCC2 A > G)* and rs6104 (*SERPINB2 C > G)* were significantly associated with PFI in the fully-adjusted model (p = 0.037 and p = 0.040, respectively), but these associations were not statistically significant in model adjusted for age only (p = 0.402 and p = 0.219, respectively). No significant correlations were found for OS.

None of the observed associations with platinum-free interval survived correction for multiple testing.

## Discussion

We correlated paclitaxel- and carboplatin-induced toxicity with genetic variation in genes involved in pharmacokinetics of these chemotherapeutics or DNA repair, and observed various correlations supporting a role for these genes in mediating toxicity and therapy outcome. We observed that several variants correlated either with grade ≥3 anemia or thrombocytopenia, use of CSFs or ESAs, as well as the platinum-free interval (see Table 5 for an overview of all significant associations). These variants are located in genes that play an important role in transport (*ABCB1*, *ABCC2*, *ABCC1*, *ABCA1*) and hepatic metabolism (*CYP3A4*) of paclitaxel and base-excision repair of platinum-induced DNA damage (*ERCC1*, *ERCC2*), thus confirming a role for these genes in mediating side-effects and efficacy of paclitaxel and carboplatin. Indeed, ATP-binding cassette transporters, which are expressed on the cell-membrane surface, play an important role in the transport of taxanes [39], whereas cytochrome P450 proteins, CYP2C8, CYP3A4 and CYP3A5, catalyze the oxidative metabolism of taxanes. Furthermore, ERCC1 and ERCC2 are subunits of the endonuclease complex that plays an essential role in DNA repair by removing platinum-induced intra-strand cross-links.

**Table 5:** **Overview of all significant correlations per genetic variant**

**Gene**	**Variant allele (rs number, nucleotide)**		**Effect of the variant allele on toxicity or outcome**	**Corrected p value, adjusted OR, 95% CI**	**Effect of the polymorphisms according to the literature**
**ABCB1**	**rs1128503**	c.1236C > T	Increased risk anemia grade 3-4	p = 0.023;	Homozygous mutant allele carriers: decreased doctaxel clearance in 92 patients ^9^
				OR 1.71, 1.07 – 2.71	
**ABCC2**	**rs12762549**	g.101620771C > G	Decreased risk anemia grade 3-4	p = 0.004;	Japanese mutant allele carriers: increased risk for severe neutropenia during treatment with docetaxel in 84 patients^19^
				OR 0.51, 0.33-0.81	
	**rs2073337**	c.1668 + 148A > G	Decreased need for colony stimulating factor	p = 0.039;	In vitro evidence: paclitaxel is a substrate of ABCC2^24^
				OR 0.60, 0.37-0.98	
**ABCC1**	**rs2074087**	c.2284-30 G > C	Increased need for erythropoiesis stimulating agent	p = 0.011;	In vitro evidence: resistance to paclitaxel with ABCC1 overexpression^23^
				OR 2.09, 1.18-3.68	
**ABCA1**	**rs363717**	c.1896 A > G	Increased risk anemia grade 3-4	p = 0.002;	Mutant allele carriers: decreased risk to develop thalidomide related neuropathy grade ≥2 in 1495 patients ^36^
				OR 2.08, 1.32-3.27	
**CYP3A4**	**rs4986910**	c.1331 T > C	Increased risk thrombocytopenia grade 3-4	p = 0.025;	-
				OR 4.99, 1.22-20.31	
**GSTP1**	**rs1695**	c.313A > G	Decreased need for colony stimulating factor	p = 0.017;	Mutant allele carriers: decreased oxaliplatin-related neuropathy in 90 patients^30^, decreased docetaxel-induced grade 2 neuropathy in 58 patients^31^, decreased risk of hematologic toxicity in 118 patients^15^
				OR 0.55, 0.33-0.90	Heterozygous mutant allele carriers compared to homozygous wildtype allele carriers: decreased PFS following cisplatin-gemcitabine in 104 patients ^32^
**ERCC1**	**rs11615**	c.354 T > C	Increased risk anemia grade 3-4	p = 0.031;	Mutant allele carriers: decreased platinum resistance in 60 patients^34^
				OR 1.61, 1.04-2.50	Heterozygous variant allele carriers compared to homozygous wildtype allele carriers: increased risk on severe neutropenia and increased likelihood of overall survival following cisplatin-gemcitabine in 104 patients^32^
**ERCC2**	**rs1799793**	c.934G > A	Decreased need for colony stimulating factor	p = 0.042;	Heterozygous variant allele carriers compared to homozygous wildtype allele carriers: increased severity of neutropenia following cisplatin-cyclophosphamide in 104 patients^32^
				OR 0.63, 0.41-0.98	
			Decreased platinum free interval	p = 0.016,	
				HR = 0.75, 0.60-0.95	

Most other studies assessing similar correlations have been performed in smaller populations, and typically evaluated only few variants. Our study evaluates a more systematically-selected panel of 26 variants in a large population of ovarian cancer patients, of which 290 were evaluable for hematologic toxicity and 265 for neurotoxicity. Another strength of our study is the availability of more detailed clinical toxicity data compared to previous pharmacogenetic association studies in ovarian cancer, allowing us to correlate specific entities of the hematologic toxicity spectrum, whereas other studies mostly grouped all hematologic > grade 3 toxicities into a single group [8,12,14,15] or focused on the occurrence of neutropenia alone [11,18].

Several of the previously published studies investigating the role of these variants with respect to toxicity and chemotherapy outcome confirmed the observations made in the present study. With respect to the ABC transporters, the rs1128503 (1236C > T) synonymous variant in *ABCB1* has been associated with multidrug resistance in multiple studies, and with decreased docetaxel clearance in particular, for homozygous carriers of the variant T-allele in 92 patients [9]. However, its association with severe anemia observed in our study has not been reported before. The rs1045642 (3435C > T) synonymous variant in *ABCB1* increased 3′p-hydroxy-paclitaxel metabolites in 23 ovarian cancer patients carrying the T-allele [8]. *Vice versa*, in a study of 26 patients, a significant greater percent decrease in absolute neutrophil count at nadir was reported for patients homozygous for the T-allele [11], Bergmann also reported a more pronounced neutrophil decrease in patients carrying the T-allele in 92 ovarian cancer patients carrying the T-allele. [18]. In our study, we failed to observe an association with grade ≥3 neutropenia or febrile neutropenia. Possibly, this is due to the fact that we analyzed grade ≥3 neutropenia whilst previous studies used absolute neutrophil decrease. A Japanese study demonstrated that carriers of the variant allele for rs12762549 (ABCC2,101620771 C > G) had an increased risk to develop docetaxel-induced leukopenia/neutropenia in 84 patients [19]. In the current study, no such association was found, but we did find a significant association with anemia and PFI, thereby confirming the potential importance of this variant in mediating taxane transport. Notably, another variant in this gene, rs2073337 (1668 + 148A > G), was significantly correlated with CSF use. For rs363717 (1896 A > G) in *ABCA1*, which was selected based on its association with thalidomide-related peripheral neuropathy [36], we did not observe a significant association with sensory neurotoxicity. We observed, however, a significant association of this variant with severe anemia (p = 0.001), suggesting that ABCA1 is involved in the transport and metabolism of platinum or carboplatin, similar to its role in the transport of cholesterol [40].

With respect to the CYP genes, low CYP3A4 enzyme activity increased the conversion of paclitaxel towards its metabolite, while heterozygous patients for CYP2C8*3 had a lower clearance of paclitaxel, suggesting the role of those genes in paclitaxel pharmacokinetics in a study of 38 patients [14]. In 93 patients with ovarian cancer, Bergmann *et al.* observed an 11% reduction in paclitaxel clearance in carriers of the rs10509681 (1196A > G) variant G-allele in *CYP2C8* [16], whereas Leskelä *et* al. observed a correlation between neurotoxicity and these *CYP2C8* and *CYP3A5* variants in a study consisting of 118 patients [25]. In the present study, we failed however, to observe such associations. Another large study in ovarian cancer also failed to observe correlations with neurotoxicity for these variants in docetaxel or paclitaxel-treated patients [12]. We did observe, however, a significant association between rs4986910 (1331 T > C) in *CYP3A4* and thrombocytopenia. Homozygous carriers of the rs1695 (313A > G) variant G-allele in *GSTP1* have been associated with neuropathy in 90 patients receiving oxaliplatin-based chemotherapy [30]. This association was not confirmed in our study, although a correlation with febrile neutropenia and CSF use was observed.

Finally, with respect to the excision repair genes, previous studies reported a correlation between severe neutropenia and the rs1799793 (934G > A) variant A-allele in *ERCC2* in 104 ovarian cancer patients receiving a cisplatin-cyclophosphamide regimen [32]. In the current study, no correlation with severe neutropenia was described, although the rs1799793 (934G > A) variant A-allele did correlate significantly with CSF use during treatment. Additionally, we observed an improved PFI for rs1799793 (934G > A) GG-carriers. The largest study to date exploring the association between 27 selected variants and ovarian cancer survival, which was performed by the ovarian cancer association consortium (OCAC) in >10,000 cases [41], rs1799793 (934G > A) was not tested. Nevertheless, in the high-grade serous sub-population of this large study, a significant correlation was found with another variant in *ERCC2* (rs50872 A > G) and outcome, confirming the potential importance of ERCC2 in mediating chemotherapy outcome. Unfortunately, rs50872 was not linked with rs1799793 (934G > A) (r^2^ = 0.06), indicating that these variants represent different association signals with *ERCC2*.

In summary, we observed the strongest associations between variants in ABC-transporters and anemia. The mechanism explaining why altered transport of cytotoxic chemotherapy affects erythropoiesis rather than granulopoiesis or thrombopoiesis is not yet understood. Possibly, these variants alter intracellular concentrations of the transported cytotoxic drug in a cell type-specific manner. Another possibility is that some cell types might be more sensitive to specific changes in the concentration of certain metabolites. On the other hand, it is also possible that the effect on erythropoiesis is caused by a specific role of the affected gene during erythropoiesis. For example, a prominent role for ABCB6 during erythropoiesis as a mitochondrial porphyrin transporter essential for heme biosynthesis, has been established [42].

It is a limitation of the current study that the group for hematologic toxicity analysis included both single agent carboplatin as paclitaxel/carboplatin combination therapy although it is known that both regimens have a slightly different hematologic toxicity profile with more thrombocytopenia in carboplatin monotherapy compared to combination regimens, in our cohort the rate of grade 3–4 thrombocytopenia was 28% for carboplatin versus 18% for combination therapy. Apart from the fact that both regimens are included for hematologic analysis, this cohort is relatively homogenous including only data on first-line treatment in ovarian cancer patients with a uniform ethnicity (99% Caucasians), high number of optimal debulked patients and relatively uniform number of administered cycles of chemotherapy. To further reduce the problem of heterogeneity, pharmacogenetic research on prospective clinical trials including large populations of uniformly treated patients is warranted.

It should be noted that the candidate-gene approach employed so far selecting drug-related genes derived from platinum/taxane pharmacology only allows the analysis with candidate genes known to be involved in chemotherapy metabolism, transport or DNA repair. To discover *novel* genetic markers, other approaches such as whole-genome association studies or targeted re-sequencing of strong candidate genes to identify rare genetic variants, could be applied. In addition, other drug- or toxicity -related candidate genes relevant for paclitaxel-carboplatin treatment in ovarian cancer (such as GSTA-1 [32], MAD1L1 [43], OPRM1 [44], TRPV1 [44], …) could be selected based on pharmacogenetic knowledge bases such as pharmGKB (www.pharmgkb.org).

## Conclusions

The current study revealed a correlation between SNPs in genes involved in DNA repair or metabolism or transport of taxanes or platinum and toxicity or response to first-line chemotherapy in ovarian cancer, using a candidate-gene approach. Variants reported in this study may serve as biomarkers and contribute to the clinical decision-making of chemotherapy dose reductions, feasibility of chemotherapy in patients at-risk based on age and/or performance status, and use of supportive medication such as ESA or CSF. However, as none of the identified associations survived correction for multiple testing, our data are only hypothesis-generating and still need independent validation. We plan to perform such a validation by performing genome-wide screens or targeted re-sequencing of candidate genes in a large multi-centered clinical trial.

## Additional files

Additional file 1: Figure S1. Study design for pharmacogenetic analyses. Additional file 2: Table S1. Minor allele frequencies of the significant SNPs, calculated for all included patients (n = 322).

## Abbreviations

SNP: Single nucleotide polymorphisms
DNA: Deoxyribonucleic acid
OR: Odds ratio
CI: Confidence interval
HR: Hazard Ratio
ATP: Adenosine 5′-triphosphate
ABCA1: ATP-binding cassette sub-family A, member 1
ABCB1: ATP-binding cassette, sub-family B, member 1
ABCC1: ATP-binding cassette, sub-family C, member 1
ABCC2: ATP-binding cassette sub-family C, member 2
ABCG2: ATP-binding cassette sub-familiy G, member 2
GSTP1: Glutathione S-transferase, pi 1
GSTT1: Glutathione S-transferase theta-1
GSTM1: Glutathione S-transferase mu-1
CYP3A4: Cytochrome P450, 3A4
CYP3A5: Cytochrome P450, 3A5
CYP2C8: Cytochrome P450, 2C8
CYP1B1: Cytochrome P450, 1B1
ERCC1: Excision repair cross-complementation group 1
ERCC2: Excision repair cross-complementation group 2
XRCC1: X-ray repair cross-complementing protein 1
SLCO1B3: Solute carrier organic anion transporter family member 1B3
SLC12A6: Solute carrier family 12 member 6
MAPT: Microtubule-associated protein tau
TUBB: Tubulin, beta class I
TP53: Tumor protein p53
SERPINB2: Serpin Peptidase Inhibitor, Clade B (Ovalbumin), Member 2
PPARD: Peroxisome Proliferator-Activated Receptor Delta
ICAM 1: Intercellular Adhesion Molecule 1
CSF: Colony stimulating factor
ESA: Erythropoiesis stimulating agent
BGOG: Belgian and Luxembourg Gynaecologic Oncology Group (BGOG)
FIGO: International Federation of Gynecology and Obstetrics
CT: Computed tomography
CA 125: Cancer antigen 125
CTCAE: Common Terminology for Adverse Events
PFI: Platinum-free interval
OS: Overall survival
AUC: Area under the curve
OC: Ovarian Cancer
NSCLC: Non-small cell lung carcinoma
FDR: False discovery rate
