# Supplementary material for: Genetic variability in drug transport, metabolism or DNA repair affecting toxicity of chemotherapy in ovarian cancer
Source: BMC Pharmacol Toxicol. 2015 Feb 27;16:2. doi: 10.1186/s40360-015-0001-5 (PMC4359565; doi:10.1186/s40360-015-0001-5)
Supplement: Additional file 1: Figure S1. — Study design for pharmacogenetic analyses. [file 40360_2015_1_MOESM1_ESM.pptx]

## Slide 1
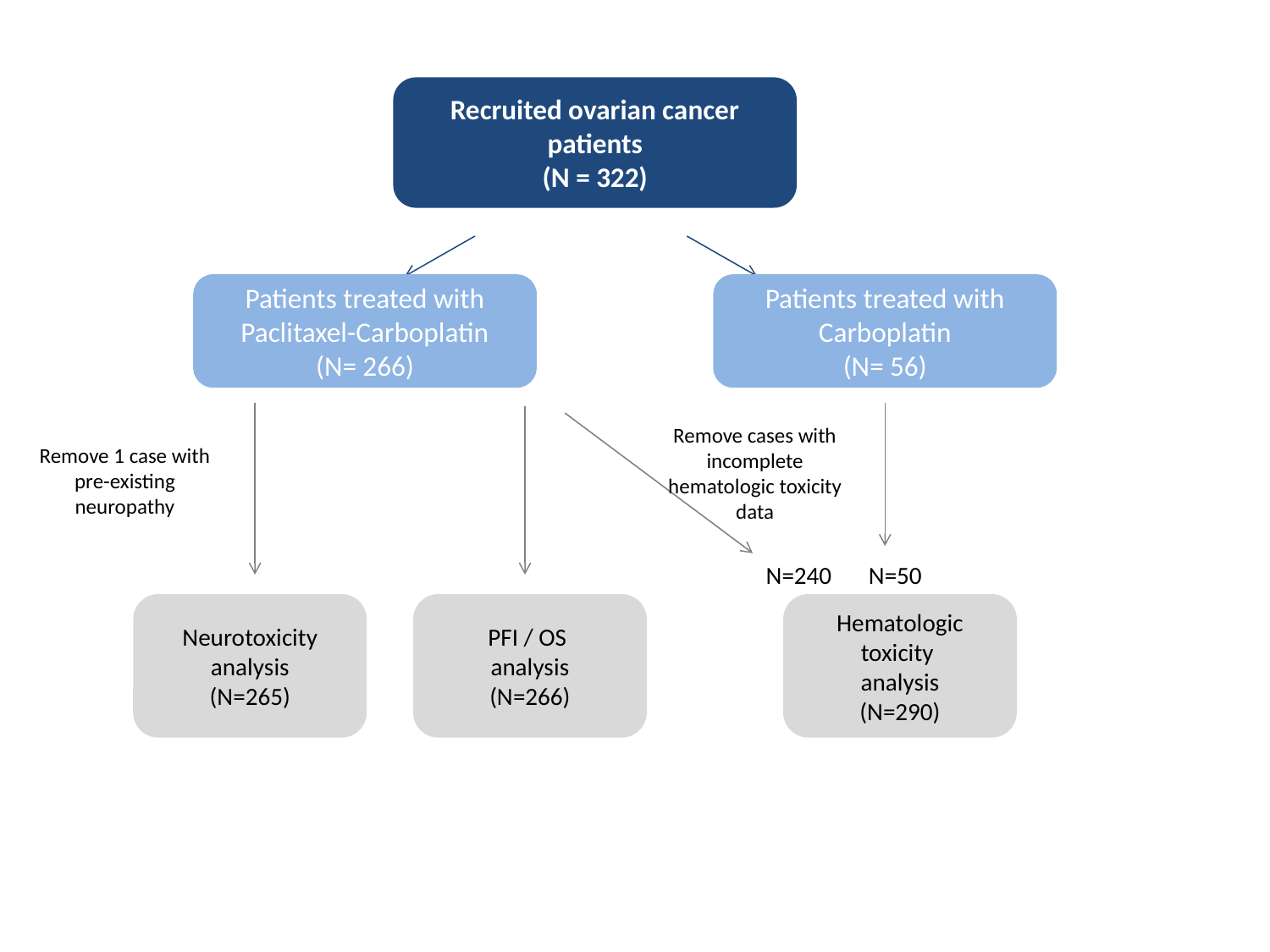

Recruited ovarian cancer patients
(N = 322)
Patients treated with Paclitaxel-Carboplatin
(N= 266)
Patients treated with Carboplatin
(N= 56)
Remove cases with incomplete hematologic toxicity data
Remove 1 case with pre-existing neuropathy
N=240
N=50
Neurotoxicity analysis
(N=265)
PFI / OS
analysis
(N=266)
Hematologic
toxicity
analysis
(N=290)
