# Supplementary material for: Genetic variability in drug transport, metabolism or DNA repair affecting toxicity of chemotherapy in ovarian cancer
Source: BMC Pharmacol Toxicol. 2015 Feb 27;16:2. doi: 10.1186/s40360-015-0001-5 (PMC4359565; doi:10.1186/s40360-015-0001-5)
Supplement: Additional file 2: Table S1. — Minor allele frequencies of the significant SNPs, calculated for all included patients (n = 322). [file 40360_2015_1_MOESM2_ESM.doc]

## Additional file 2: Table S1: Minor allele frequencies of the significant SNPs, calculated for all included patients (n=322)

| **Gene** | **Single nucleotide polymorphism** | | **Hoz**  **wt** | **Hez** | **Hoz**  **m** | **Mis** | **Minor allele** | **MAF** | **HWE**  **p-value** |
| --- | --- | --- | --- | --- | --- | --- | --- | --- | --- |
| **ABCB1** | rs1128503 | c.1236C>T | 102 | 164 | 56 | 0 | T | 0.428 | 0.474 |
| **ABCC2** | rs12762549 | g.101620771C>G | 93 | 146 | 81 | 2 | G | 0.481 | 0.123 |
|  | rs2073337 | c.1668+148A>G | 118 | 159 | 45 | 0 | G | 0.387 | 0.461 |
| **ABCC1** | rs2074087 | c.2284-30G>C | 237 | 77 | 7 | 1 | C | 0.142 | 0.801 |
| **ABCA1** | rs363717 | c.1683-5676A>G | 92 | 144 | 86 | 0 | G | 0.491 | 0.059 |
| **CYP3A4** | rs4986910 | c.1331T>C | 312 | 9 | 0 | 1 | C | 0.014 | 0.799 |
| **GSTP1** | rs1695 | c.313A>G | 134 | 154 | 34 | 0 | G | 0.345 | 0.292 |
| **ERCC1** | rs11615 | c.354T>C | 146 | 130 | 45 | 1 | C | 0.343 | 0.070 |
| **ERCC2** | rs1799793 | c.934G>A | 150 | 124 | 40 | 8 | A | 0.325 | 0.077 |

**Legend to table S1:** Hoz wt: homozygous wildtype, Hez: heterozygous variant, Hoz v: homozygous mutant, Mis: missing value, MAF: minor allele frequencies, HWE: Hardy-Weinberg equilibrium
